# Co-expression of transcription factor AP-2beta (*TFAP2B*) and GATA3 in human mammary epithelial cells with *intense, apicobasal* immunoreactivity for CK8/18

**DOI:** 10.1007/s10735-021-09980-2

**Published:** 2021-06-11

**Authors:** M. Raap, L. Gierendt, C. Werlein, E. Kuehnle, H. H. Kreipe, M. Christgen

**Affiliations:** 1 Institute of Pathology, Hannover Medical School, Carl-Neuberg-Str. 1, 30625 Hannover, Germany; 2 Department of Gynecology and Obstetrics, Hannover Medical School, Hannover, Germany

**Keywords:** AP-2β, *TFAP2B*, Normal breast, GATA3, CK8/18

## Abstract

AP-2β is a new mammary epithelial differentiation marker and its expression is preferentially retained and enhanced in lobular carcinoma in situ and invasive lobular breast cancer. In normal breast epithelium AP-2β is expressed in a scattered subpopulation of luminal cells. So far, these cells have not been further characterized. Co-expression of AP-2β protein and luminal epithelium markers (GATA3, CK8/18), hormone receptors [estrogen receptor (ER), androgen receptor (AR)] and candidate stem cells markers (CK5/14, CD44) were assessed by double-immunofluorescence staining in normal mammary gland epithelium. The subpopulation of AP-2β-positive mammary epithelial cells showed an almost complete, superimposable co-expression with GATA3 and a peculiar intense, ring-like appearing immunoreactivity for CK8/18. Confocal immunofluorescence microscopy revealed an apicobasal staining for CK8/18 in AP-2β-positive cells, which was not seen in in AP-2β-negative cells. Furthermore, AP-2β-positive displayed a partial co-expression with ER and AR, but lacked expression of candidate stem cell markers CK5/14 and CD44. In summary, AP-2β is a new luminal mammary epithelial differentiation marker, which is expressed in the GATA3-positive subpopulation of luminal epithelial cells. These AP-2β-positive/GATA3-positive cells also show a peculiar CK8/18-expression which may indicate a previously unknown functionally specialized mammary epithelial cell population.

**Supplementary Information:**

The online version contains supplementary material available at 10.1007/s10735-021-09980-2.

## Introduction

*TFAP2B*/AP-2β is a member of the activator protein-2 (AP-2) family of transcription factors, which comprises five members (AP-2α, -2β, -2γ, -2δ, -2ε), encoded by separate genes (*TFAP2A*, *TFAP2B*, *TFAP2C*, *TFAP2D*, *TFAP2E*) (Pellikainen and Kosma 2007). AP-2 proteins bind GC-rich DNA sequences and mediate both activating and repressive stimuli. AP-2 proteins function in a cell type-specific manner and regulate embryonic organ development, differentiation and tissue homeostasis. During embryogenesis, AP-2α, AP-2β and AP-2γ display partially overlapping expression patterns in the nervous system, the facial mesenchyme, the limbs and various epithelia (Martino et al. 2016; Moser et al. 1997; Pellikainen and Kosma 2007; Seki et al. 2015). AP-2δ and AP-2ε are restricted to the central nervous system (Hesse et al. 2011).

The human *TFAP2B* gene is primarily known for its association with the Char syndrome, an autosomal dominant disorder characterized by patent ductus arteriosus, facial dysmorphism and anatomical abnormalities of the fifth digit (Satoda et al. 2000). More recently, *TFAP2B* has been implicated in neoplastic diseases (Ebauer et al. 2007; Grass et al. 2009; Ikram et al. 2016; Li et al. 2018; Wachtel et al. 2006). *AP-2β* is overexpressed in alveolar rhabdomyosarcoma (aRMS), a rare childhood malignancy (Wachtel et al. 2006). Contrary to the results in aRMS, AP-2β seems to be a favorable prognostic marker in carcinomas such as endometrial cancer (Wu and Zhang 2018), cervical cancer (Wang et al. 2017), renal cell cancer (Oya et al. 2004), neuroblastoma (Ikram et al. 2016; Thorell et al. 2009) and breast cancer (BC) (Raap et al. 2018; Yoldi et al. 2016). Regarding AP-2 transcription factors in the mammary gland and breast cancer, most studies have focused on *TFAP2A*/AP-2α and *TFAP2C*/AP-2γ (Friedrichs et al. 2005; Gee et al. 2009; Orso et al. 2004; Pellikainen et al. 2002; Shiu et al. 2014; Turner et al. 1998; Williams et al. 2009). However, various prognostic BC gene expression signatures, such as the 496 intrinsic gene set, include *TFAP2B* as a classifier gene (Guedj et al. 2012; Hu et al. 2006; Korkola et al. 2003; Perou et al. 2000; Weigelt et al. 2010). Moreover, we have recently observed, that AP-2β-positivity is associated with favorable clinicopathologic factors such as a positive estrogen receptor (ER) and androgen receptor (AR) status and low Ki67 (Raap et al. 2018). Also AP-2β is associated with a prolonged event-free survival of BC patients and with the lobular BC subtype and its precursor lesion lobular carcinoma in situ (LCIS) (Raap et al. 2018). AP-2β expression can be observed in normal breast epithelium in a cell population with a nuclear localization between the luminal and the myoepithelial cell compartment (Raap et al. 2018). This localization is different from the reported pattern of AP-2α (entire luminal cell layer) and AP-2γ (entire myoepithelial cell layer) (Friedrichs et al. 2005). Fridriksdottir et al. showed AP-2β-expression in the ER-positive and estrogen-responsive epithelial cell compartment in normal human breast (Fridriksdottir et al. 2015). Furthermore, various reactive and metaplastic mammary gland lesions show enhanced AP-2β expression in terms of an expansion of the AP-2β-positive cell population (Raap et al. 2018). The peculiar AP-2β-staining pattern, the expression in non-neoplastic cell proliferations and the enhanced expression in ILBC and LCIS prompted us to further investigate the characteristics of AP-2β-positive cells in normal mammary epithelium, by analyzing the co-expression of AP-2β with luminal mammary epithelial markers (GATA3 (Chou et al. 2010), CK8/18 (Boecker et al. 2018)), hormone receptors (ER, AR) and candidate stem cell markers (CK5/14 (Boecker et al. 2018), CD44(Fillmore and Kuperwasser 2007)).

## Methods

### Patient characteristics

Formalin-fixed paraffin-embedded (FFPE) breast tissue specimens from 11 female patients were retrieved from the tissue archive of the Institute of Pathology of the Hannover Medical School (MHH) according to the guidelines of the local ethics committee. All specimens were made anonymous. Breast reduction surgery was the type of surgery performed in all 11 patients. Two age-groups of patients were chosen. The age of the younger, presumably pre-menopausal patients was 21–27 (n = 5). The age of the older, presumably peri-/post-menopausal patients was 58–67 (n = 6) (Table 1). Information about hormonal factors and the menstrual cycle were not available. One patient with fibroadenoma and one patient with contralateral breast cancer but without systemic therapy prior to breast reduction were included. All cases were re-reviewed on HE-stained full sections to confirm normal lobular and ductular configuration and exclude neoplastic, metaplastic or inflammatory changes. One representative tissue block from each patient was chosen for further analysis.

**Table 1:** Patient characteristics

	Age	Indication
Patient 1	27	Breast reduction
Patient 2	24	Breast reduction
Patient 3	21	Breast reduction
Patient 4	22	Breast reduction
Patient 5	23	Breast reduction^a^
Patient 6	67	Breast reduction
Patient 7	67	Breast reduction^b^
Patient 8	61	Breast reduction
Patient 9	58	Breast reduction
Patient 10	65	Breast reduction
Patient 11	66	Breast reduction

^a^With contralateral breast cancer

^b^With fibroadenoma

### Immunohistochemistry, double-immunofluorescence and confocal microscopy

For antigen-detection 4 µm sections of FFPE tissue blocks were mounted on superfrost slides (Thermo Fisher Scientific, Waltham, MA, U.S.A.). Slides were deparaffinized and rehydrated conventionally. Immunohistochemistry staining was performed on a Benchmark Ultra (Ventana, Tucson, AZ, U.S.A.). Antibodies and staining protocols are detailed in Online Resource 1.

For double-immunofluorescence, different staining protocols were applied depending on the antibodies. For the rabbit polyclonal anti-ER antibody (1:100, Clone H-184, Santa Cruz Biotechnology, Dallas, TX, U.S.A.) slides were incubated with the primary antibody for 1 h at 37 °C. After washing, slides were incubated with secondary goat anti-rabbit antibody labeled with Cy3. After blocking with donkey-anti-goat serum for 30 min at 37 °C the second staining with the rabbit polyclonal anti-AP-2β antibody H-87 (1:50, Santa Cruz Biotechnology) was performed for 1 h at 37 °C, followed by incubation with the secondary goat anti-rabbit antibody labeled with Cy2 or AlexaFluor488 for confocal microscopy. Specificity and sensitivity of the H-87 antibody was confirmed previously (Raap et al. 2018). For mouse antibodies the slides were incubated with the rabbit polyclonal anti-AP-2β antibody H-87 (1:50) and the mouse monoclonal antibodies anti-AR (1:50, Clone R441, Dako, Glostrup, Denmark), anti-CK5/14 (1:200, Clone XM26 + LL002, Diagnostics BioSystems, The Hague, Netherlands), anti-CK8/18 (1:300, Clone NCL-5D3, Leica Biosystems, Newcastle upon Tyne, UK) or anti-GATA3 (1:100, Zytomed Systems, Berlin, Germany). After blocking for 5 min, slides were incubated with secondary goat anti-rabbit antibodies labeled with Cy2 and goat anti-mouse antibodies labeled with Cy3 or AlexaFluor647 for confocal microscopy. Cells were counterstained with Hoechst 33342 (1 μg/ml, Thermo Fisher Scientific). Visualization was performed using an Axio Imager Z1 fluorescence microscope (Zeiss, Oberkochen, Germany). Confocal fluorescence microscopy was performed on a Leica SP8 inverted confocal microscope (Leica microsystems, Wetzlar, Germany) using a 63 × oil immersion objective. Fluorophores were excited using a 405 nm diode for DAPI, an Argon Laser (488 nm) for AlexaFluor488 (excitation 496 nm/emission 519 nm) and a HeNe laser for AlexaFluor647 (excitation 650 nm/emission 665 nm). Images were acquired by separate sequential imaging for each fluorophore at a resolution of 1024 × 1024 pixel and a slice thickness of 0.2 µm. Maximum intensitiy projections and processing of the images were performed using FIJI (Schindelin et al. 2012).

To determine the fraction of mammary epithelial cells with co-expression of AP-2β and either of the five above mentioned markers, 100 cells in 10 spots (5 ducts, 5 lobules) of each stained slide of the 11 patients were examined. The number of cells with expression of AP-2β only, the number of cells with expression of the second markers only (ER, AR, CK8/18, GATA3, CK5/14, CD44), the number of cells with co-expression of AP-2β and these markers and the number of cells without any marker expression were documented. The luminal and myoepithelial cell layers were distinguished by morphology. Screening by eye-balling for co-expression with further markers (Additional antibodies and staining protocols are detailed in Online Resource 2) was performed on tissue of patient 5.

### Statistics

Statistical analysis of AP-2β co-expression with other immunohistochemical markers was performed using the GraphPad Prism software (version 5.00) and unpaired t-test.

## Results

### Co-expression of AP-2β and luminal epithelium markers GATA3 and CK8/18

**Fig. 1:**
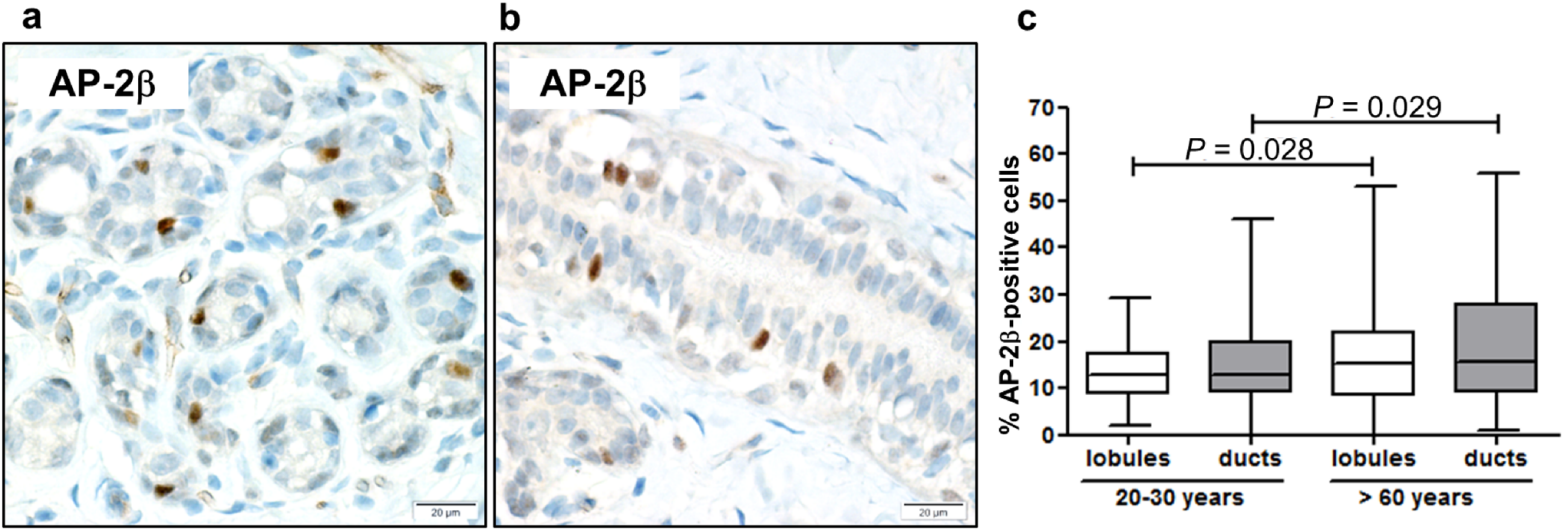
AP-2β expression in the normal mammary gland. Immunohistochemistry shows scattered AP-2β-positive cells in the epithelium of mammary gland lobules (**a**) and mammary gland ducts (**b**). **c** Analysis of double-immunofluorescence staining of 100 cells in 10 spots (5 ducts, 5 lobules) of each stained slide of 11 patients shows an expression of AP-2β in approximately 15% of mammary gland epithelial cells express with a slightly higher expression in the older patients group

**Fig. 2:**
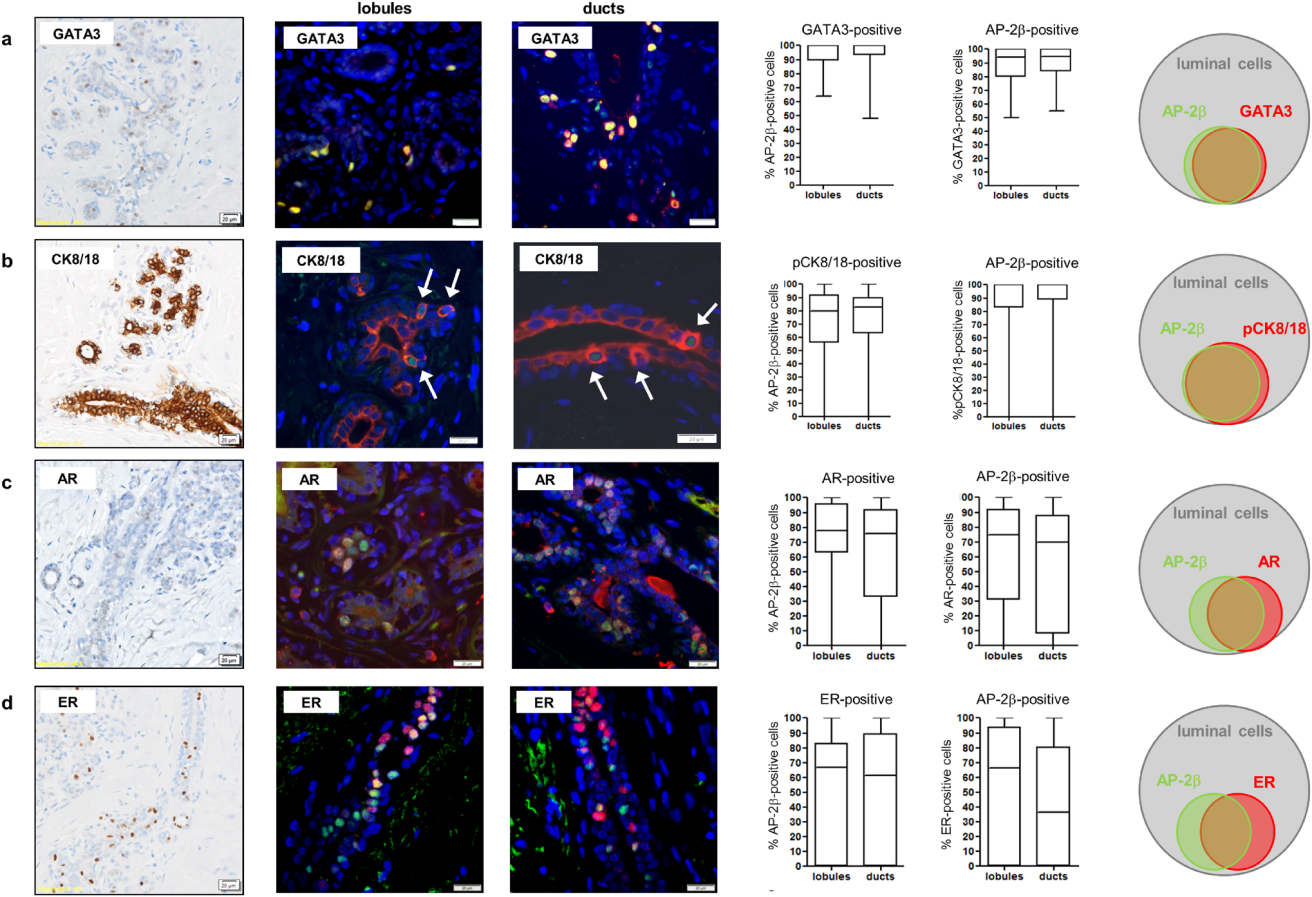
Co-expression of AP-2β with luminal epithelial markers and hormone receptors in the normal mammary gland. The first column shows immunohistochemical staining; the second and third columns show double-immunofluorescence stainings; the fourth and fifth columns show statistical results from double-immunofluorescence analysis counting 100 cells in 10 spots (5 ducts, 5 lobules) of each stained slide of 11 patients; the last column shows Venn-diagrams, visualizing the proportion of morphological identified luminal mammary epithelial cells with (co-)expression of AP-2β (green) and the depicted markers (red) **a** Results from double-immunofluorescence show an almost exclusive co-expression of AP-2β (green) and GATA3 (red). **b** Double-immunofluorescence staining for AP-2β (green) and CK8/18 (red) reveal a peculiar intense and perinuclear appearing staining pattern (white arrow, pCK8/18) of the AP-2β- positive epithelial cells in ducts and lobules (**c, d**). Double-immunofluorescence staining for AP-2β (green) and AR or ER (red) show a partial co-expression of these markers

We have previously shown, that AP-2β is expressed in approximately 30% of luminal breast epithelial cells (Raap et al. 2018). The nuclei of positively labelled cells are localized below the luminal and above the basal cell layer of ductal epithelium (Fig. 1a) (Raap et al. 2018). For further characterization of AP-2β expressing cells, double-immunofluorescence staining with the luminal epithelial markers GATA3 and CK8/18 was performed. To determine the fraction of cells with co-expression of AP-2β and the above mentioned markers, 100 cells in 10 spots (5 ducts, 5 lobules) of each stained slide of 11 patients were examined and the number of cells with and without co-expression was documented. Approximately 15% of luminal mammary lobular and ductal epithelial cells stained positive for AP-2β with a slightly higher fraction of AP-2β-positive cells in the breast tissue of the older patients group (lobular *P* = 0.028, ductal *P* = 0.029, unpaired t-test) (Fig. 1a–c). Double-immunofluorescence staining showed an almost complete co-expression of AP-2β and GATA3 (Fig. 2a). As expected, double-immunofluorescence staining with CK8/18 showed that AP-2β expression was restricted to cells expressing these luminal epithelial cytokeratins. Interestingly, in epifluorescence-microscopy the AP-2β-positive cells did not show a cytoplasmic peripheral-predominant appearing CK8/18-staining pattern as the majority of luminal epithelial cells, but a more intense cytoplasmic staining, which appeared ring-like and perinuclear (Fig. 2b, arrows). Confocal microscopy confirmed a more intense staining for CK8/18 in the AP-2β-positive cells, when compared to the AP-2β-negative cells. Furthermore, the AP-2β-positive cells showed an apicobasal staining for CK8/18, which was not seen in AP-2β-negative cells. These two staining patterns resulted in a ring-like appearance in the maximum intensity projection of all z-stacks, comparable to the staining pattern in epifluorescence-microscopy (Online Resource 3). This staining pattern was almost exclusively restricted to AP-2β-positive cells. It was reproducible with other antibodies against CK8 and CK18 (data not shown, for antibody information see Online Resource 2). No such staining pattern was found for other keratins such as CK19 and CK7 (Online Resource 4). In bright field immunohistochemical staining this intense, apicobasal and ring-like appearing staining pattern was difficult to retrace because of the high staining intensity for CK8/18 (Fig. 2b).

### Partial co-expression of AP-2β and hormone-receptors ER and AR

ER and AR are expressed by ductal and lobular epithelial cells depending on age, pregnancy status and phase of menstrual cycle but constantly only in a minority of non-neoplastic breast epithelial cells (Hallberg et al. 2008; Kensler et al. 2018; Khan et al. 2002). Our data showed a higher fraction of ER-positive cells in breast epithelium of older patients (lobular: 17 ± 9%, ductal: 16 ± 14%) compared to the younger patients (lobular: 5 ± 7%, ductal: 4 ± 7%) (lobular and ductal *P* < 0.001, unpaired t-test) (Online Resource 5). Likewise, a higher fraction of AR-positive cells was found in breast epithelium of the older patients (lobular: 18 ± 9%, ductal: 19 ± 12%) compared to the younger patients (lobular: 10 ± 9%, ductal: 8 ± 8%) (lobular *P* = 0.004, ductal *P* < 0.001, unpaired t-test) (Online Resource 5). Double-immunofluorescence staining showed a partial co-expression of AP-2β and AR. AR-positive cells showed an expression of AP-2β in a median of 78% of lobular and 76% of ductal cells. Conversely, AP-2β-positive cells showed an expression of AR in 75% of lobular and 70% of ductal cells (Fig. 2c). Double-immunofluorescence staining showed a partial co-expression of AP-2β and ER. ER-positive cells showed an expression of AP-2β in a median of 67% of lobular and 62% of ductal cells. Conversely, AP-2β-positive cells showed expression of ER in 67% of lobular and 37% of ductal cells (Fig. 2d).

### AP-2β and potential stem cell markers CK5/14 and CD44 are not co-expressed

**Fig. 3:**
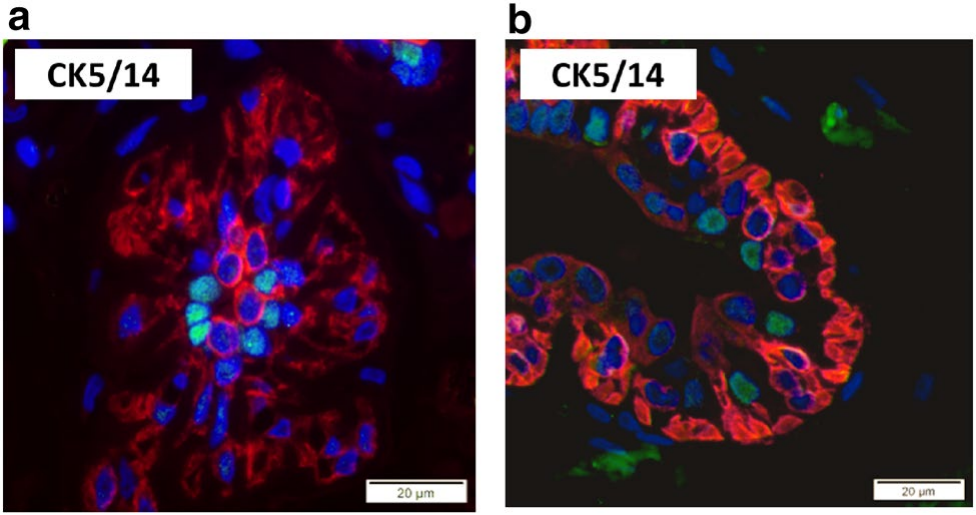
AP-2β and CK5/14 are not co-expressed in the human mammary epithelium. Double-immunofluorescence staining for AP-2β (green) and the potential stem cell marker CK5/14 (red) of a mammary gland lobule (**a**) and a mammary gland duct (**b**)

Various potential mammary epithelial stem cell markers have been suggested by earlier studies. Boecker et al. postulated that CK5/14-positive luminal cells might be breast epithelium stem-cells (Boecker et al. 2018). We observed no co-expression of AP-2β and CK5/14 (Fig. 3). Furthermore studies using fluorescence-activated cell sorting-analyses suggested high expression of CD44 in potential stem cells (Fillmore and Kuperwasser 2007). To our knowledge, an immunohistochemical identification of these cells has not been attempted so far. Using immunofluorescence staining a cell population with enhanced expression of CD44 was not readily discernable. Screening of the double-immunofluorescence staining did not identify a specific co-expression of AP-2β and CD44 (Online Resource 4).

## Discussion

AP-2β is a transcription factor which is expressed in different ectodermal, neuroectodermal and mesenchymal cells. Epithelial tissues with AP-2β expression include distal tubule epithelium of the kidney, salivary gland epithelium, the basal cell layer of the epidermis and squamous epithelium of the esophagus and mammary gland epithelium (Raap et al. 2018). In breast cancer, AP-2β is associated with favorable clinicopathologic markers such as ER- and AR-positivity and low Ki67, with prolonged event-free survival and the lobular breast cancer subtype (Raap et al. 2018). In normal breast tissue, AP-2β-positive cells show a localization of the nucleus intermediate between the luminal and basal cell compartment. This peculiar localization and the results from breast cancer studies prompted us to further characterize this cell population in normal breast epithelium (Raap et al. 2018). Samples from reduction mammoplasties were chosen for this purpose. In our study, approximately 15% of luminal mammary lobular and ductal epithelial cells stained positive for AP-2β, with a slightly higher percentage of AP-2β-positive cells in the older presumably peri-/postmenopausal patients group. The distribution and proportion of epithelial cells in mammary ducts and lobules, which express factors such as AP-2β, GATA3 and hormone receptors differ widely on an inter-individual and also intra-individual basis. Information about hormonal factors and the menstrual cycle were not available. Therefore, the percentage of positive cells and the differences between age groups, such as a slightly higher expression of AP-2β and a significant higher expression of the hormone receptors (ER, AR) in older patients, need to be interpreted with caution.

Earlier studies showed expression of AP-2β in the ER-positive and estrogen-responsive epithelial cell compartment in normal human breast (Fridriksdottir et al. 2015) and no co-expression of AP-2β and p63 (Raap et al. 2018). This leads to the conclusion, that AP-2β is expressed in luminal differentiated epithelial cells. This conclusion is underscored by our findings of co-expression of AP-2β with the luminal epithelial markers GATA3 and CK8/18 and partially with ER and AR. Comparable to AP-2β Fridriksdottir et al. showed expression of GATA3 in the ER-positive and estrogen-responsive breast epithelial cells (Fridriksdottir et al. 2015). Fridriksdottir et al. have not analysed the fraction of cells showing co-expression of AP-2β and GATA3. Our analyses have revealed an almost complete overlap of AP-2β and GATA3 expression in a luminal epithelial subpopulation. These cells show a peculiar intense, apicobasal and ring-like appearing immunoreactivity for CK8/18. This staining pattern is different from the adjacent AP-2β-negative epithelial cells, which display a weaker basolateral staining. Other keratins (CK7, CK9/19) did not show that kind of expression. Interestingly, this CK8/18-staining pattern can also be seen in images in earlier studies (Böcker et al. 2009), but to our knowledge has never been described, discussed or investigated. Unlike in normal breast tissue, a comparable CK8 staining pattern has been noticed in invasive lobular breast cancer (ILBC) and its precursor lesion lobular carcinoma in situ (LCIS) (Lehr et al. 2000). This is of interest as AP-2β expression is enhanced in ILBC and LCIS (Raap et al. 2018). A relation between the AP-2β-positive epithelial cells and lobular neoplasias could be suspected. Co-expression analyses with potential stem cell markers CK5/14 and CD44 did not provide any hint for a conceivable localization of AP-2β in a stem cell compartment. However, the concept of breast epithelium and breast cancer stem cells is widely discussed and we have chosen only two of a wide variety of supposed stem cell markers. Yoldi et al. show in their supplemental data a potential regulatory function of AP-2β on CK8 expression in breast cancer cells (Yoldi et al. 2016). Accordingly, further analyses of the influence of AP-2β on CK8/18 and GATA3 could be of interest in ILBC and in the normal mammary gland.

In summary, AP-2β is a new luminal mammary epithelial differentiation marker, which is expressed in the GATA3-positive subpopulation of luminal epithelial cells. These AP-2β-positive/GATA3-positive cells also show a peculiar intense, apicobasal and ring-like appearing CK8/18 expression which may indicate a previously unknown functionally specialized mammary epithelial cell population.

## Supplementary Information

Below is the link to the electronic supplementary material.

Supplementary Information 1 (DOCX 16 kb)

Supplementary Information 2 (DOCX 15 kb)

Supplementary Information 3 (TIF 466 kb)

Supplementary Information 4 (DOCX 20 kb)

## Data Availability

All authors declare that all data and materials published claim and comply with field standards.

## References

[CR1] Böcker W, Hungermann D, Decker T (2009). Anatomy of the breast. Pathologe.

[CR2] Boecker W, van Horn L, Stenman G, Sturken C, Schumacher U, Loening T, Liesenfeld L, Korsching E, Glaser D, Tiemann K, Buchwalow I (2018). Spatially correlated phenotyping reveals K5-positive luminal progenitor cells and p63–K5/14-positive stem cell-like cells in human breast epithelium. Lab Investig.

[CR3] Chou J, Provot S, Werb Z (2010). GATA3 in development and cancer differentiation: cells GATA have it!. J Cell Physiol.

[CR4] Ebauer M, Wachtel M, Niggli FK, Schafer BW (2007). Comparative expression profiling identifies an in vivo target gene signature with TFAP2B as a mediator of the survival function of PAX3/FKHR. Oncogene.

[CR5] Fillmore C, Kuperwasser C (2007). Human breast cancer stem cell markers CD44 and CD24: enriching for cells with functional properties in mice or in man?. Breast Cancer Res.

[CR6] Fridriksdottir AJ, Kim J, Villadsen R, Klitgaard MC, Hopkinson BM, Petersen OW, Rønnov-Jessen L (2015). Propagation of oestrogen receptor-positive and oestrogen-responsive normal human breast cells in culture. Nat Commun.

[CR7] Friedrichs N, Jager R, Paggen E, Rudlowski C, Merkelbach-Bruse S, Schorle H, Buettner R (2005). Distinct spatial expression patterns of AP-2alpha and AP-2gamma in non-neoplastic human breast and breast cancer. Mod Pathol.

[CR8] Gee JM, Eloranta JJ, Ibbitt JC, Robertson JF, Ellis IO, Williams T, Nicholson RI, Hurst HC (2009). Overexpression of TFAP2C in invasive breast cancer correlates with a poorer response to anti-hormone therapy and reduced patient survival. J Pathol.

[CR9] Grass B, Wachtel M, Behnke S, Leuschner I, Niggli FK, Schafer BW (2009). Immunohistochemical detection of EGFR, fibrillin-2, P-cadherin and AP2beta as biomarkers for rhabdomyosarcoma diagnostics. Histopathology.

[CR10] Guedj M, Marisa L, de Reynies A, Orsetti B, Schiappa R, Bibeau F, MacGrogan G, Lerebours F, Finetti P, Longy M, Bertheau P, Bertrand F, Bonnet F, Martin AL, Feugeas JP, Bieche I, Lehmann-Che J, Lidereau R, Birnbaum D, Bertucci F, de The H, Theillet C (2012). A refined molecular taxonomy of breast cancer. Oncogene.

[CR11] Hallberg G, Persson I, Naessén T, Magnusson C (2008). Effects of pre- and postmenopausal use of exogenous hormones on receptor content in normal human breast tissue: a randomized study. Gynecol Endocrinol.

[CR12] Hesse K, Vaupel K, Kurt S, Buettner R, Kirfel J, Moser M (2011). AP-2delta is a crucial transcriptional regulator of the posterior midbrain. PLoS ONE.

[CR13] Hu Z, Fan C, Oh DS, Marron JS, He X, Qaqish BF, Livasy C, Carey LA, Reynolds E, Dressler L, Nobel A, Parker J, Ewend MG, Sawyer LR, Wu J, Liu Y, Nanda R, Tretiakova M, Ruiz Orrico A, Dreher D, Palazzo JP, Perreard L, Nelson E, Mone M, Hansen H, Mullins M, Quackenbush JF, Ellis MJ, Olopade OI, Bernard PS, Perou CM (2006). The molecular portraits of breast tumors are conserved across microarray platforms. BMC Genomics.

[CR14] Ikram F, Ackermann S, Kahlert Y, Volland R, Roels F, Engesser A, Hertwig F, Kocak H, Hero B, Dreidax D, Henrich KO, Berthold F, Nurnberg P, Westermann F, Fischer M (2016). Transcription factor activating protein 2 beta (TFAP2B) mediates noradrenergic neuronal differentiation in neuroblastoma. Mol Oncol.

[CR15] Kensler KH, Beca F, Baker GM, Heng YJ, Beck AH, Schnitt SJ, Hazra A, Rosner BA, Eliassen AH, Hankinson SE, Brown M, Tamimi RM (2018). Androgen receptor expression in normal breast tissue and subsequent breast cancer risk. NPJ Breast Cancer.

[CR16] Khan SA, Yee KA, Kaplan C, Siddiqui JF (2002). Estrogen receptor alpha expression in normal human breast epithelium is consistent over time. Int J Cancer.

[CR17] Korkola JE, DeVries S, Fridlyand J, Hwang ES, Estep AL, Chen YY, Chew KL, Dairkee SH, Jensen RM, Waldman FM (2003). Differentiation of lobular versus ductal breast carcinomas by expression microarray analysis. Cancer Res.

[CR18] Lehr HA, Folpe A, Yaziji H, Kommoss F, Gown AM (2000). Cytokeratin 8 immunostaining pattern and E-cadherin expression distinguish lobular from ductal breast carcinoma. Am J Clin Pathol.

[CR19] Li Z, Xu X, Luo M, Hao J, Zhao S, Yu W, Xiao X, Wu J, Zheng F, Chen M, Li Y, Qin G, Liao Y, Zhao X, Yu X, Guo W, Zou L, Deng W (2018). Activator protein-2beta promotes tumor growth and predicts poor prognosis in breast cancer. Cell Physiol Biochem.

[CR20] Martino VB, Sabljic T, Deschamps P, Green RM, Akula M, Peacock E, Ball A, Williams T, West-Mays JA (2016). Conditional deletion of AP-2beta in mouse cranial neural crest results in anterior segment dysgenesis and early-onset glaucoma. Dis Model Mech.

[CR21] Moser M, Ruschoff J, Buettner R (1997). Comparative analysis of AP-2 alpha and AP-2 beta gene expression during murine embryogenesis. Dev Dyn.

[CR22] Orso F, Cottone E, Hasleton MD, Ibbitt JC, Sismondi P, Hurst HC, De Bortoli M (2004). Activator protein-2gamma (AP-2gamma) expression is specifically induced by oestrogens through binding of the oestrogen receptor to a canonical element within the 5'-untranslated region. Biochem J.

[CR23] Oya M, Mikami S, Mizuno R, Miyajima A, Horiguchi Y, Nakashima J, Marumo K, Mukai M, Murai M (2004). Differential expression of activator protein-2 isoforms in renal cell carcinoma. Urology.

[CR24] Pellikainen J, Kataja V, Ropponen K, Kellokoski J, Pietilainen T, Bohm J, Eskelinen M, Kosma VM (2002). Reduced nuclear expression of transcription factor AP-2 associates with aggressive breast cancer. Clin Cancer Res.

[CR25] Pellikainen JM, Kosma VM (2007). Activator protein-2 in carcinogenesis with a special reference to breast cancer–a mini review. Int J Cancer.

[CR26] Perou CM, Sorlie T, Eisen MB, van de Rijn M, Jeffrey SS, Rees CA, Pollack JR, Ross DT, Johnsen H, Akslen LA, Fluge O, Pergamenschikov A, Williams C, Zhu SX, Lonning PE, Borresen-Dale AL, Brown PO, Botstein D (2000). Molecular portraits of human breast tumours. Nature.

[CR27] Raap M, Gronewold M, Christgen H, Glage S, Bentires-Alj M, Koren S, Derksen PW, Boelens M, Jonkers J, Lehmann U, Feuerhake F, Kuehnle E, Gluz O, Kates R, Nitz U, Harbeck N, Kreipe HH, Christgen M (2018). Lobular carcinoma in situ and invasive lobular breast cancer are characterized by enhanced expression of transcription factor AP-2beta. Lab Investig.

[CR28] Satoda M, Zhao F, Diaz GA, Burn J, Goodship J, Davidson HR, Pierpont ME, Gelb BD (2000). Mutations in TFAP2B cause Char syndrome, a familial form of patent ductus arteriosus. Nat Genet.

[CR29] Schindelin J, Arganda-Carreras I, Frise E, Kaynig V, Longair M, Pietzsch T, Preibisch S, Rueden C, Saalfeld S, Schmid B, Tinevez JY, White DJ, Hartenstein V, Eliceiri K, Tomancak P, Cardona A (2012). Fiji: an open-source platform for biological-image analysis. Nat Methods.

[CR30] Seki R, Kitajima K, Matsubara H, Suzuki T, Saito D, Yokoyama H, Tamura K (2015). AP-2beta is a transcriptional regulator for determination of digit length in tetrapods. Dev Biol.

[CR31] Shiu KK, Wetterskog D, Mackay A, Natrajan R, Lambros M, Sims D, Bajrami I, Brough R, Frankum J, Sharpe R, Marchio C, Horlings H, Reyal F, van der Vijver M, Turner N, Reis-Filho JS, Lord CJ, Ashworth A (2014). Integrative molecular and functional profiling of ERBB2-amplified breast cancers identifies new genetic dependencies. Oncogene.

[CR32] Thorell K, Bergman A, Caren H, Nilsson S, Kogner P, Martinsson T, Abel F (2009). Verification of genes differentially expressed in neuroblastoma tumours: a study of potential tumour suppressor genes. BMC Med Genomics.

[CR33] Turner BC, Zhang J, Gumbs AA, Maher MG, Kaplan L, Carter D, Glazer PM, Hurst HC, Haffty BG, Williams T (1998). Expression of AP-2 transcription factors in human breast cancer correlates with the regulation of multiple growth factor signalling pathways. Cancer Res.

[CR34] Wachtel M, Runge T, Leuschner I, Stegmaier S, Koscielniak E, Treuner J, Odermatt B, Behnke S, Niggli FK, Schafer BW (2006). Subtype and prognostic classification of rhabdomyosarcoma by immunohistochemistry. J Clin Oncol.

[CR35] Wang F, Huang W, Hu X, Chen C, Li X, Qiu J, Liang Z, Zhang J, Li L, Wang X, Ding X, Xiang S, Zhang J (2017). Transcription factor AP-2beta suppresses cervical cancer cell proliferation by promoting the degradation of its interaction partner beta-catenin. Mol Carcinog.

[CR36] Weigelt B, Geyer FC, Natrajan R, Lopez-Garcia MA, Ahmad AS, Savage K, Kreike B, Reis-Filho JS (2010). The molecular underpinning of lobular histological growth pattern: a genome-wide transcriptomic analysis of invasive lobular carcinomas and grade- and molecular subtype-matched invasive ductal carcinomas of no special type. J Pathol.

[CR37] Williams CM, Scibetta AG, Friedrich JK, Canosa M, Berlato C, Moss CH, Hurst HC (2009). AP-2gamma promotes proliferation in breast tumour cells by direct repression of the CDKN1A gene. EMBO J.

[CR38] Wu H, Zhang J (2018). Decreased expression of TFAP2B in endometrial cancer predicts poor prognosis: A study based on TCGA data. Gynecol Oncol.

[CR39] Yoldi G, Pellegrini P, Trinidad EM, Cordero A, Gomez-Miragaya J, Serra-Musach J, Dougall WC, Munoz P, Pujana MA, Planelles L, Gonzalez-Suarez E (2016). RANK signaling blockade reduces breast cancer recurrence by inducing tumor cell differentiation. Cancer Res.

