# Supplementary material for: Co-expression of transcription factor AP-2beta (TFAP2B) and GATA3 in human mammary epithelial cells with intense, apicobasal immunoreactivity for CK8/18
Source: J Mol Histol. 2021 Jun 11;52(6):1257–64. doi: 10.1007/s10735-021-09980-2 (PMC8616868; doi:10.1007/s10735-021-09980-2)
Supplement: Supplementary file 1 — Supplementary Information 1 (DOCX 16 kb) [file 10735_2021_9980_MOESM1_ESM.docx]

| Supplemental Data Table 1 | |  |  |  | |  |  |
| --- | --- | --- | --- | --- | --- | --- | --- |
| Antibodies used for immunohistochemical analysis | | | | | | |  |
| antigen | antibody | species | source | | dilution | antigenic retrieval | detection system |
| AP-2beta  AR  CK5/14  CK8/18  ER  GATA-3 | clone H-87  clone AR441  clones XM26 & LL002  clone 5D3  clone SP1  clone L50-823 | rabbit  mouse  mouse  mouse  rabbit  mouse | Santa Cruz Biotechnology  Dako  Zytomed  Leica Biosystems  Ventana  Biocare medical | | 1:250  1:50  1:200  1:100  undiluted, ready-to use solution  1:100 | CC1 mild protocol (Ventana)  CC1 mild protocol (Ventana)  CC1 mild protocol (Ventana)  CC1 mild protocol (Ventana)  CC1 mild protocol (Ventana)  CC1 mild protocol (Ventana) | ultraView DAB Kit (Ventana)  ultraView DAB Kit (Ventana)  ultraView DAB Kit (Ventana)  ultraView DAB Kit (Ventana)  ultraView DAB Kit (Ventana)  ultraView DAB Kit (Ventana) |

“Co-expression of Transcription Factor AP-2beta(TFAP2B) and GATA3 in human mammary epithelial cells with perinuclear immunoreactivity for CK8/18”, Journal of Molecular Histology, M. Raap, L.Gierendt, T.W. Park-Simon, H.H. Kreipe, M. Christgen, Correspondance to Mieke Raap (Institute of Pathology, Hannover Medical School, Hannover, Germany, [Raap.Mieke@MH-Hannover.de](mailto:Raap.Mieke@MH-Hannover.de))
