# Supplementary material for: Co-expression of transcription factor AP-2beta (TFAP2B) and GATA3 in human mammary epithelial cells with intense, apicobasal immunoreactivity for CK8/18
Source: J Mol Histol. 2021 Jun 11;52(6):1257–64. doi: 10.1007/s10735-021-09980-2 (PMC8616868; doi:10.1007/s10735-021-09980-2)
Supplement: Supplementary file 2 — Supplementary Information 2 (DOCX 15 kb) [file 10735_2021_9980_MOESM2_ESM.docx]

“Co-expression of Transcription Factor AP-2beta(TFAP2B) and GATA3 in human mammary epithelial cells with perinuclear immunoreactivity for CK8/18”, Journal of Molecular Histology, M. Raap, L.Gierendt, T.W. Park-Simon, H.H. Kreipe, M. Christgen, Correspondance to Mieke Raap (Institute of Pathology, Hannover Medical School, Hannover, Germany, [Raap.Mieke@MH-Hannover.de](mailto:Raap.Mieke@MH-Hannover.de))

| Supplemental Data Table 2 | |  |  |  |  |  |  |  |
| --- | --- | --- | --- | --- | --- | --- | --- | --- |
| Additional antibodies used for double-immunofluorescence analysis | | | | | |  |  |  |
| antigen | antibody | species | source | dilution |  |  |  |  |
| CD44  CK7  CK8  CK18  CK19 | clone DF1485  clone OV-TL 12/30  clone TS1  clone DC-10  clone A53-B/2.26 | mouse  mouse  mouse  mouse  mouse | Dako  Dako  Leica Biosystems  Novocastra  Zytomed | 1:50  1:300  1:100  1:100  1:100 |  |  |  |  |
|  |  |  |  |  |  |  |  |  |
