# Supplementary figures and images for: Co-expression of transcription factor AP-2beta (TFAP2B) and GATA3 in human mammary epithelial cells with intense, apicobasal immunoreactivity for CK8/18
Source: J Mol Histol. 2021 Jun 11;52(6):1257–64. doi: 10.1007/s10735-021-09980-2 (PMC8616868; doi:10.1007/s10735-021-09980-2)

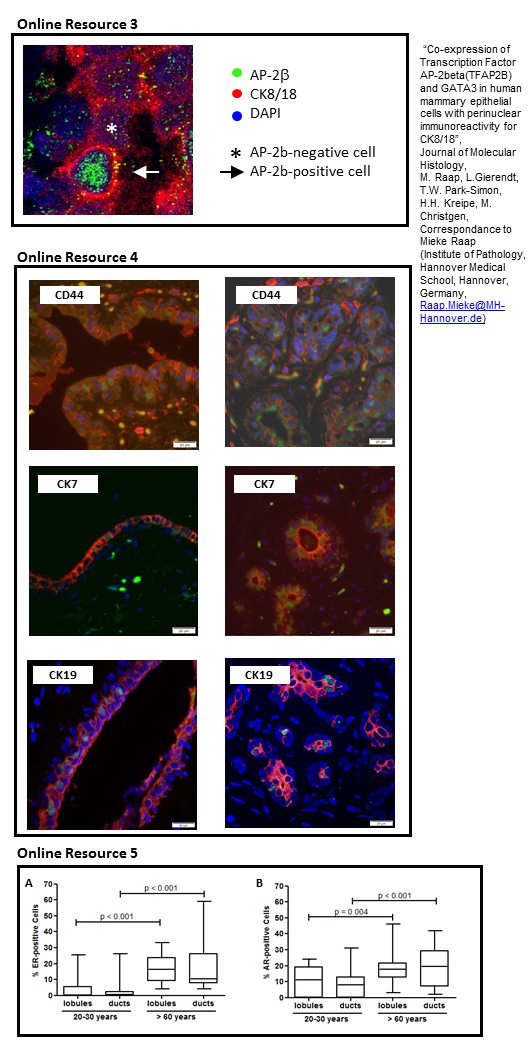

Supplement: Supplementary file 3 — Supplementary Information 3 (TIF 466 kb) [file 10735_2021_9980_MOESM3_ESM.tif]
