# Supplementary material for: Co-expression of transcription factor AP-2beta (TFAP2B) and GATA3 in human mammary epithelial cells with intense, apicobasal immunoreactivity for CK8/18
Source: J Mol Histol. 2021 Jun 11;52(6):1257–64. doi: 10.1007/s10735-021-09980-2 (PMC8616868; doi:10.1007/s10735-021-09980-2)
Supplement: Supplementary file 4 — Supplementary Information 4 (DOCX 20 kb) [file 10735_2021_9980_MOESM4_ESM.docx]

# List of supplemental data online

**Online Resource 1** Table: Antibodies used for immunohistochemical staining

**Online Resource 2** Table: Additional antibodies used for double- immunofluorescence staining

**Online Resource 3** Figure: Confocal imaging of AP-2β and CK8/18

**Online Resource 4** Figure: Double‑immunofluorescence staining of AP-2β, CD44, CK7 and CK19

**Online Resource 5** Figure: Increased fraction of ER- and AR-positive normal mammary epithelial cells in the older patients cohort
